# Bis(tetra­ethyl­ammonium) bis­(hydrogen l-tartrate) l-tartaric acid monohydrate

**DOI:** 10.1107/S1600536811015479

**Published:** 2011-05-07

**Authors:** M. Rajalakshmi, R. Indirajith, R. Gopalakrishnan, K. Ramamurthi, Helen Stoeckli-Evans

**Affiliations:** a Department of Physics, Anna University, Chennai 600 025, India; b Crystal Growth and Thin Film Laboratory, School of Physics, Bharathidasan University, Tiruchirappalli 620 024, India; c Institute of Physics, University of Neuchâtel, CH-2000 Neuchâtel, Switzerland

## Abstract

In the title compound, 2C_8_H_20_N^+^·2C_4_H_5_O_6_^−^·C_4_H_6_O_6_·H_2_O, the presence of the two tetra­ethyl­ammonium cations is balanced by two hydrogen l-tartrate anions. Also present in the asymmetric unit are a mol­ecule of l-tartaric acid and a water mol­ecule. The various components are linked by O—H⋯O hydrogen bonds. In the crystal, two-dimensional networks are formed *via* O—H⋯O hydrogen bonds and C—H⋯O inter­actions involving the water mol­ecule, the hydrogen l-tartrate anions and the l-tartaric acid mol­ecules. These layers, which stack along [001], are separated by tetra­ethyl­ammonium cations. The latter are also involved in C—H⋯O inter­actions with the anions and the l-tartaric acid and water mol­ecules participating in the two-dimensional network.

## Related literature

For potential industrial applications of non-linear optical (NLO) materials, see: Dega-Szafran *et al.* (2008 ▶); Bosshard *et al.* (1995 ▶). For an example of a structure showing bulk quadratic NLO effects, see: Coe *et al.* (2005 ▶). For the crystal structure of tetra­ethyl­ammonium hydrogen l-tartrate dihydrate, see: Rahman *et al.* (2008 ▶).
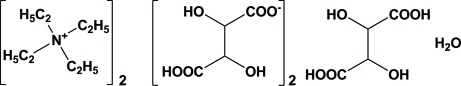


## Experimental

### 

#### Crystal data

2C_8_H_20_N^+^·2C_4_H_5_O_6_^−^·C_4_H_6_O_6_·H_2_O
*M* *_r_* = 726.76
Monoclinic, 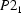

*a* = 7.5725 (4) Å
*b* = 27.7907 (13) Å
*c* = 8.7620 (6) Å
β = 99.884 (5)°
*V* = 1816.55 (18) Å^3^
*Z* = 2
Mo *K*α radiation
μ = 0.11 mm^−1^
*T* = 173 K
0.45 × 0.32 × 0.25 mm

#### Data collection

Stoe IPDS 2 diffractometer
20250 measured reflections
3502 independent reflections
3116 reflections with *I* > 2σ(*I*)
*R*_int_ = 0.072

#### Refinement

*R*[*F* ^2^ > 2σ(*F* ^2^)] = 0.032
*wR*(*F* ^2^) = 0.064
*S* = 1.03
3502 reflections
469 parameters
1 restraint
H atoms treated by a mixture of independent and constrained refinement
Δρ_max_ = 0.17 e Å^−3^
Δρ_min_ = −0.20 e Å^−3^

### 

Data collection: *X-AREA* (Stoe & Cie, 2009 ▶); cell refinement: *X-AREA*; data reduction: *X-RED32* (Stoe & Cie, 2009 ▶); program(s) used to solve structure: *SHELXS97* (Sheldrick, 2008 ▶); program(s) used to refine structure: *SHELXL97* (Sheldrick, 2008 ▶); molecular graphics: *PLATON* (Spek, 2009 ▶) and *Mercury* (Macrae *et al.*, 2006 ▶); software used to prepare material for publication: *SHELXL97* and *PLATON* .

## Supplementary Material

Crystal structure: contains datablocks I, global. DOI: 10.1107/S1600536811015479/nr2003sup1.cif

Structure factors: contains datablocks I. DOI: 10.1107/S1600536811015479/nr2003Isup2.hkl

Additional supplementary materials:  crystallographic information; 3D view; checkCIF report

## Figures and Tables

**Table 1:** Hydrogen-bond geometry (Å, °)

*D*—H⋯*A*	*D*—H	H⋯*A*	*D*⋯*A*	*D*—H⋯*A*
O1*W*—H1*WB*⋯O2	0.89 (5)	2.56 (4)	2.995 (3)	111 (3)
O1*W*—H1*WB*⋯O5^i^	0.89 (5)	2.19 (5)	3.015 (3)	155 (4)
O3—H3*O*⋯O1*W*	0.84	2.04	2.868 (3)	167
O4—H4*O*⋯O11	0.84	1.99	2.790 (2)	160
O6—H6*O*⋯O2^ii^	0.84	1.65	2.485 (2)	175
O9—H9*O*⋯O1*W*^ii^	0.84	2.25	3.077 (3)	169
O10—H10*O*⋯O5	0.84	2.05	2.848 (2)	158
O14—H14*O*⋯O7^iii^	0.84	1.73	2.552 (2)	164
O15—H15*O*⋯O1	0.84	2.05	2.869 (2)	166
O16—H16*O*⋯O12^iii^	0.84	2.27	3.054 (3)	156
O18—H18*O*⋯O1^ii^	0.84	1.77	2.588 (2)	165
C3—H3⋯O15	1.00	2.55	3.503 (3)	158
C7—H7⋯O13^iv^	1.00	2.52	3.396 (3)	146
C13—H13*A*⋯O1*W*^v^	0.99	2.51	3.419 (4)	152
C16—H16*B*⋯O3^vi^	0.98	2.46	3.378 (3)	155
C19—H19*A*⋯O3^vi^	0.99	2.38	3.275 (3)	150
C19—H19*B*⋯O9^vi^	0.99	2.37	3.294 (3)	155
C23—H23*A*⋯O17^vii^	0.99	2.47	3.420 (3)	161
C25—H25*A*⋯O13	0.99	2.46	3.419 (3)	164
C26—H26*A*⋯O9^viii^	0.98	2.56	3.482 (4)	157
C27—H27*B*⋯O18^i^	0.99	2.49	3.180 (3)	126

Symmetry codes: (i) 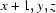; (ii) 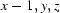; (iii) 
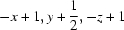
; (iv) 
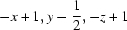
; (v) 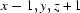; (vi) 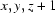; (vii) 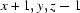; (viii) 
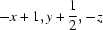
.

**Fig. 1.**
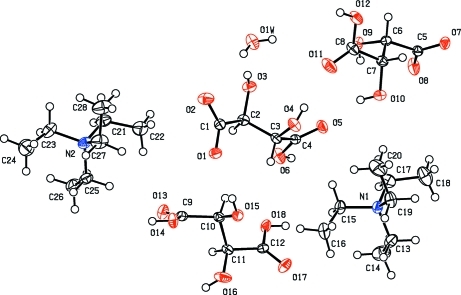
The molecular structure of the title compound with displacement ellipsoids drawn at the 50% probability level.

**Fig. 2.**
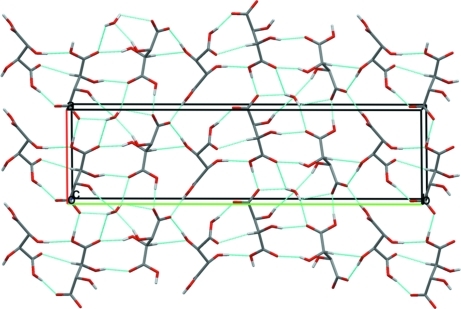
A view along the c axis of the crystal packing of the title compound, with the O—H···O hydrogen bonds shown as dashed cyan lines (see Table 1 for details; C-bound H-atoms and the Tetra-ethylammonium cations have been omitted for clarity).

**Fig. 3.**
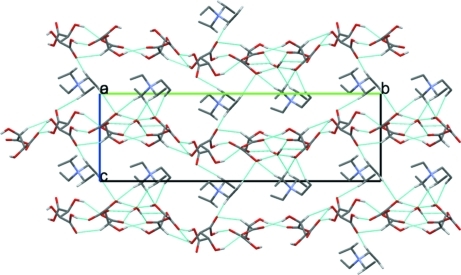
A view along the a axis of the crystal packing of the title compound, showing both the O—H···O hydrogen bonds and the C—H···O interactions as dashed cyan lines (see Table 1 for details; C-bound H-atoms not involved in the C—H···O interactions have been omitted for clarity).

## supplementary crystallographic information

### Comment

Our interest in the determination of the structure of the title compound is due to recent advances in organic non-linear optical (NLO) materials on account of their widespread potential industrial applications (Dega-Szafran *et al.*, 2008; Bosshard *et al.*, 1995). The majority of promising compounds constitute dipolar donor-π-acceptor molecules and these must be arranged non-centrosymmetrically to afford macroscopic structures capable of showing bulk quadratic NLO effects, such as frequency doubling [second-harmonic generation, SHG] (Coe *et al.*, 2005). In particular, molecule-based NLO materials offer ultrafast response times, lower dielectric constants, better processability characteristics and enhanced nonresonant NLO responses relative to the traditional inorganic crystals. Previous work has shown that an inherent relationship exists between the structure of title material and its observed properties, although the SHG output was found to be rather weak when compared to KDP or urea.

The molecular structure of the title compound is illustrated in Fig. 1. The asymmetric unit is composed of two tetraethylammonium cations, two hydrogen L-tartrate anions, a molecule of L-tartaric acid and a water molecule. The various moieties are linked by O—H···O hydrogen bonds (Table 1).

In the crystal two-dimensional networks (Fig. 2) are formed *via* O—H···O hydrogen bonds and C—H···O interactions (Table 1) involving the water molecule, the hydrogen L-tartrate anions and the L-tartaric acid molecules. These layers stack along [001] are separated by tetraethylammonium cations, which are also involved in C—H···O interactions with the anions and the L-tartaric acid and water molecules (Fig. 3 and Table 1). This arrangement is similar to that in the crystal structure of Tetra-ethylammonium hydrogen L-tartrate dihydrate, which has been reported on previously (Rahman *et al.*, 2008).

### Experimental

The title compound was synthesized using tetraethyl ammonium and L-tartaric acid in an equimolar ratio. The measured quantity of L-tartaric was dissolved in double distilled water until a saturated solution was obtained. Tetraethylammonium hydroxide (20% water) was then added slowly drop wise to the aqueous solution of L-tartaric acid. The mixture was stirred well at RT until a homogeneous solution was obtained. It was then stirred for 4 hrs at 350 K (oil bath) and then cooled to RT. The cooled solution was then filtered and the filtrate covered using a thick parafilm sheet, in order to control the evaporation rate at RT in a constant temperature bath. Good quality single crystals of title compound were obtained after 1 month.

### Refinement

In the final cycles of refinement, in the absence of significant anomalous scattering effects, 3324 Friedel pairs were merged and Δf " set to zero. The absolute configuration is referred to that of L-tartaric acid. The water H-atoms were located in difference electron-density maps and were refined with O—H distance restraints of 0.84 (2) Å, and *U*_iso_(H) = 1.5 × *U*_eq_(O). The OH and C-bound H-atoms were included in calculated positions and treated as riding atoms: O—H = 0.84 Å, C—H = 1.0, 0.99 and 0.98Å for CH, CH_2_, and CH_3_ H-atoms, respectively, with *U*_iso_(H) = k × *U*_eq_(C or O), where k = 1.5 for OH and CH_3_ H-atoms, and k = 1.2 for all other H-atoms.

### Crystal data

**Table:**

2C_8_H_20_N^+^·2C_4_H_5_O_6_^−^·C_4_H_6_O_6_·H_2_O	*F*(000) = 784
*M**_r_* = 726.76	*D*_x_ = 1.329 Mg m^−^^3^
Monoclinic, *P*2_1_	Mo *K*α radiation, λ = 0.71073 Å
Hall symbol: P 2yb	Cell parameters from 16128 reflections
*a* = 7.5725 (4) Å	θ = 1.5–25.7°
*b* = 27.7907 (13) Å	µ = 0.11 mm^−^^1^
*c* = 8.7620 (6) Å	*T* = 173 K
β = 99.884 (5)°	Rod, colourless
*V* = 1816.55 (18) Å^3^	0.45 × 0.32 × 0.25 mm
*Z* = 2	

### Data collection

**Table:**

Stoe IPDS 2 diffractometer	3116 reflections with *I* > 2σ(*I*)
Radiation source: fine-focus sealed tube	*R*_int_ = 0.072
graphite	θ_max_ = 25.7°, θ_min_ = 1.5°
φ and ω scans	*h* = −9→9
20250 measured reflections	*k* = −33→33
3502 independent reflections	*l* = −10→10

### Refinement

**Table:**

Refinement on *F*^2^	Secondary atom site location: difference Fourier map
Least-squares matrix: full	Hydrogen site location: inferred from neighbouring sites
*R*[*F*^2^ > 2σ(*F*^2^)] = 0.032	H atoms treated by a mixture of independent and constrained refinement
*wR*(*F*^2^) = 0.064	*w* = 1/[σ^2^(*F*_o_^2^) + (0.0336*P*)^2^] where *P* = (*F*_o_^2^ + 2*F*_c_^2^)/3
*S* = 1.03	(Δ/σ)_max_ < 0.001
3502 reflections	Δρ_max_ = 0.17 e Å^−^^3^
469 parameters	Δρ_min_ = −0.20 e Å^−^^3^
1 restraint	Extinction correction: *SHELXL97* (Sheldrick, 2008), Fc^*^=kFc[1+0.001xFc^2^λ^3^/sin(2θ)]^-1/4^
Primary atom site location: structure-invariant direct methods	Extinction coefficient: 0.0045 (10)

### Special details

**Table:**

Geometry. All e.s.d.'s (except the e.s.d. in the dihedral angle between two l.s. planes) are estimated using the full covariance matrix. The cell e.s.d.'s are taken into account individually in the estimation of e.s.d.'s in distances, angles and torsion angles; correlations between e.s.d.'s in cell parameters are only used when they are defined by crystal symmetry. An approximate (isotropic) treatment of cell e.s.d.'s is used for estimating e.s.d.'s involving l.s. planes.
Refinement. The water H-atoms were located in difference electron-density maps and refined with O—H distance restraints of 0.84 (2) Å, and *U*_iso_(H) = 1.5 × *U*_eq_(O). The OH and C-bound H-atoms were included in calculated positions and treated as riding atoms: O—H = 0.84 Å, C—H = 1.0, 0.99 and 0.98Å for CH, CH_2_, and CH_3_ H-atoms, respectively, with *U*_iso_(H) = k × *U*_eq_(C or O), where k = 1.5 for OH and CH_3_ H-atoms, and k = 1.2 for all other H-atoms.

### Fractional atomic coordinates and isotropic or equivalent isotropic displacement parameters (Å^2^)

**Table:**

	*x*	*y*	*z*	*U*_iso_*/*U*_eq_	
O1	0.8300 (2)	0.28144 (6)	0.5505 (2)	0.0269 (4)	
O2	0.9206 (2)	0.23806 (7)	0.3631 (2)	0.0337 (5)	
O3	0.6051 (2)	0.19685 (6)	0.27276 (19)	0.0251 (4)	
H3O	0.7027	0.1818	0.2838	0.038*	
O4	0.6211 (2)	0.17495 (6)	0.5971 (2)	0.0255 (4)	
H4O	0.5879	0.1502	0.5453	0.038*	
O5	0.2590 (2)	0.16831 (6)	0.4724 (2)	0.0267 (4)	
O6	0.2518 (2)	0.24709 (6)	0.4224 (2)	0.0314 (4)	
H6O	0.1406	0.2424	0.4023	0.047*	
C1	0.8051 (3)	0.25215 (8)	0.4390 (3)	0.0199 (5)	
C2	0.6153 (3)	0.23299 (9)	0.3861 (3)	0.0194 (5)	
H2	0.5407	0.2606	0.3384	0.023*	
C3	0.5318 (3)	0.21533 (8)	0.5243 (3)	0.0188 (5)	
H3	0.5441	0.2419	0.6022	0.023*	
C4	0.3328 (3)	0.20709 (9)	0.4694 (3)	0.0199 (5)	
O7	0.0108 (2)	−0.03787 (6)	0.4425 (2)	0.0231 (4)	
O8	−0.1122 (2)	0.02964 (6)	0.3374 (2)	0.0342 (5)	
O9	0.1768 (2)	0.05712 (6)	0.2309 (2)	0.0256 (4)	
H9O	0.0916	0.0746	0.2475	0.038*	
O10	0.2592 (2)	0.07004 (6)	0.5647 (2)	0.0267 (4)	
H10O	0.2803	0.0963	0.5242	0.040*	
O11	0.5811 (3)	0.08248 (6)	0.4720 (3)	0.0391 (5)	
O12	0.5832 (2)	0.00705 (6)	0.3876 (3)	0.0342 (5)	
H12O	0.6874	0.0144	0.3751	0.051*	
C5	0.0186 (3)	0.00157 (8)	0.3764 (3)	0.0182 (5)	
C6	0.1979 (3)	0.01833 (8)	0.3365 (3)	0.0187 (5)	
H6	0.2516	−0.0092	0.2870	0.022*	
C7	0.3276 (3)	0.03191 (8)	0.4858 (3)	0.0204 (5)	
H7	0.3407	0.0032	0.5556	0.025*	
C8	0.5108 (3)	0.04356 (9)	0.4477 (3)	0.0239 (6)	
O13	0.5634 (2)	0.41652 (7)	0.4106 (2)	0.0291 (4)	
O14	0.7719 (2)	0.39729 (6)	0.6164 (2)	0.0266 (4)	
H14O	0.8314	0.4188	0.5809	0.040*	
O15	0.5743 (2)	0.32771 (6)	0.70813 (19)	0.0222 (4)	
H15O	0.6599	0.3135	0.6779	0.033*	
O16	0.4137 (3)	0.41234 (7)	0.7886 (2)	0.0358 (5)	
H16O	0.4190	0.4418	0.7683	0.054*	
O17	0.1584 (2)	0.34469 (7)	0.80706 (19)	0.0307 (4)	
O18	0.1062 (2)	0.33623 (6)	0.54942 (19)	0.0244 (4)	
H18O	0.0235	0.3180	0.5672	0.037*	
C9	0.6136 (3)	0.39366 (8)	0.5289 (3)	0.0203 (5)	
C10	0.4884 (3)	0.35829 (8)	0.5892 (3)	0.0189 (5)	
H10	0.4295	0.3379	0.5009	0.023*	
C11	0.3428 (3)	0.38693 (8)	0.6524 (3)	0.0199 (5)	
H11	0.2910	0.4109	0.5719	0.024*	
C12	0.1928 (3)	0.35356 (8)	0.6810 (3)	0.0178 (5)	
N1	0.1498 (3)	0.18045 (7)	0.9192 (2)	0.0216 (4)	
C13	0.0283 (4)	0.20263 (11)	1.0211 (3)	0.0311 (6)	
H13A	−0.0298	0.1763	1.0704	0.037*	
H13B	0.1034	0.2210	1.1049	0.037*	
C14	−0.1157 (4)	0.23552 (13)	0.9396 (4)	0.0462 (8)	
H14C	−0.1862	0.2482	1.0146	0.069*	
H14B	−0.1944	0.2175	0.8589	0.069*	
H14A	−0.0603	0.2623	0.8923	0.069*	
C15	0.2489 (4)	0.21890 (9)	0.8449 (3)	0.0262 (6)	
H15A	0.3226	0.2030	0.7765	0.031*	
H15B	0.1597	0.2393	0.7788	0.031*	
C16	0.3694 (4)	0.25100 (10)	0.9570 (3)	0.0343 (6)	
H16C	0.4319	0.2736	0.8991	0.052*	
H16B	0.4574	0.2312	1.0245	0.052*	
H16A	0.2969	0.2689	1.0202	0.052*	
C17	0.0424 (4)	0.15163 (10)	0.7866 (3)	0.0304 (6)	
H17A	−0.0401	0.1739	0.7209	0.036*	
H17B	0.1265	0.1386	0.7221	0.036*	
C18	−0.0661 (4)	0.11051 (11)	0.8345 (4)	0.0457 (8)	
H18C	−0.1327	0.0949	0.7419	0.069*	
H18B	−0.1505	0.1228	0.8982	0.069*	
H18A	0.0144	0.0871	0.8944	0.069*	
C19	0.2818 (4)	0.14840 (10)	1.0239 (3)	0.0301 (6)	
H19A	0.3407	0.1679	1.1129	0.036*	
H19B	0.2134	0.1226	1.0655	0.036*	
C20	0.4256 (4)	0.12536 (12)	0.9483 (4)	0.0461 (8)	
H20A	0.5018	0.1505	0.9153	0.069*	
H20B	0.3695	0.1067	0.8579	0.069*	
H20C	0.4987	0.1040	1.0226	0.069*	
N2	0.9558 (3)	0.42249 (8)	0.1066 (2)	0.0252 (5)	
C21	0.8261 (4)	0.38890 (10)	0.0071 (3)	0.0318 (6)	
H21A	0.7490	0.4082	−0.0730	0.038*	
H21B	0.8958	0.3664	−0.0470	0.038*	
C22	0.7068 (5)	0.35979 (12)	0.0949 (4)	0.0492 (8)	
H22C	0.6219	0.3409	0.0214	0.074*	
H22B	0.6407	0.3816	0.1526	0.074*	
H22A	0.7809	0.3380	0.1673	0.074*	
C23	1.0666 (4)	0.44630 (11)	−0.0008 (3)	0.0335 (6)	
H23A	1.1215	0.4208	−0.0562	0.040*	
H23B	0.9852	0.4651	−0.0793	0.040*	
C24	1.2140 (5)	0.47937 (12)	0.0767 (5)	0.0510 (9)	
H24C	1.2734	0.4943	−0.0022	0.076*	
H24B	1.3017	0.4607	0.1484	0.076*	
H24A	1.1622	0.5044	0.1342	0.076*	
C25	0.8563 (4)	0.45954 (9)	0.1868 (3)	0.0282 (6)	
H25A	0.7907	0.4426	0.2591	0.034*	
H25B	0.9455	0.4808	0.2494	0.034*	
C26	0.7250 (4)	0.49045 (10)	0.0805 (4)	0.0374 (7)	
H26C	0.6702	0.5138	0.1421	0.056*	
H26B	0.6315	0.4700	0.0222	0.056*	
H26A	0.7881	0.5076	0.0083	0.056*	
C27	1.0750 (4)	0.39489 (11)	0.2343 (3)	0.0377 (7)	
H27A	1.1613	0.4176	0.2936	0.045*	
H27B	0.9995	0.3818	0.3062	0.045*	
C28	1.1785 (5)	0.35398 (12)	0.1782 (3)	0.0440 (8)	
H28C	1.2500	0.3378	0.2673	0.066*	
H28B	1.2580	0.3666	0.1106	0.066*	
H28A	1.0945	0.3309	0.1204	0.066*	
O1W	0.9034 (3)	0.13191 (8)	0.3037 (3)	0.0361 (5)	
H1WA	0.873 (5)	0.1078 (16)	0.348 (5)	0.060 (12)*	
H1WB	0.985 (6)	0.1475 (16)	0.370 (5)	0.069 (13)*	

### Atomic displacement parameters (Å^2^)

**Table:**

	*U*^11^	*U*^22^	*U*^33^	*U*^12^	*U*^13^	*U*^23^
O1	0.0211 (9)	0.0305 (9)	0.0308 (10)	−0.0086 (8)	0.0089 (8)	−0.0131 (8)
O2	0.0154 (9)	0.0414 (11)	0.0452 (12)	−0.0024 (8)	0.0077 (8)	−0.0189 (9)
O3	0.0180 (9)	0.0331 (10)	0.0232 (9)	−0.0021 (7)	0.0009 (7)	−0.0088 (8)
O4	0.0278 (10)	0.0189 (8)	0.0260 (9)	−0.0017 (8)	−0.0057 (8)	0.0017 (7)
O5	0.0242 (10)	0.0206 (9)	0.0354 (10)	−0.0068 (8)	0.0060 (8)	−0.0020 (8)
O6	0.0118 (9)	0.0243 (9)	0.0581 (13)	−0.0007 (8)	0.0065 (9)	0.0040 (9)
C1	0.0165 (12)	0.0195 (11)	0.0235 (12)	0.0017 (10)	0.0029 (10)	0.0003 (10)
C2	0.0140 (12)	0.0211 (12)	0.0222 (12)	0.0026 (10)	0.0004 (10)	−0.0013 (10)
C3	0.0174 (12)	0.0149 (11)	0.0238 (12)	−0.0002 (10)	0.0025 (10)	−0.0017 (10)
C4	0.0205 (12)	0.0186 (12)	0.0219 (12)	−0.0012 (11)	0.0074 (10)	−0.0040 (10)
O7	0.0187 (9)	0.0225 (9)	0.0298 (9)	0.0009 (7)	0.0090 (7)	0.0041 (7)
O8	0.0159 (9)	0.0255 (9)	0.0626 (14)	0.0045 (8)	0.0109 (9)	0.0130 (9)
O9	0.0228 (10)	0.0293 (9)	0.0260 (9)	0.0013 (8)	0.0082 (8)	0.0094 (7)
O10	0.0301 (10)	0.0242 (9)	0.0279 (9)	0.0003 (8)	0.0115 (8)	−0.0056 (8)
O11	0.0245 (10)	0.0228 (10)	0.0701 (15)	−0.0060 (8)	0.0082 (10)	−0.0118 (9)
O12	0.0177 (10)	0.0198 (9)	0.0682 (14)	−0.0026 (8)	0.0163 (9)	−0.0094 (9)
C5	0.0158 (12)	0.0180 (12)	0.0207 (12)	−0.0014 (10)	0.0030 (9)	−0.0023 (10)
C6	0.0169 (13)	0.0173 (11)	0.0229 (12)	0.0022 (10)	0.0065 (10)	−0.0003 (10)
C7	0.0191 (13)	0.0185 (11)	0.0234 (13)	0.0022 (9)	0.0027 (10)	−0.0011 (9)
C8	0.0158 (12)	0.0194 (13)	0.0347 (15)	−0.0002 (10)	−0.0009 (11)	−0.0013 (11)
O13	0.0290 (10)	0.0348 (10)	0.0243 (10)	−0.0056 (8)	0.0066 (8)	0.0061 (8)
O14	0.0192 (9)	0.0259 (9)	0.0342 (10)	−0.0075 (7)	0.0032 (8)	0.0052 (8)
O15	0.0215 (9)	0.0196 (8)	0.0266 (9)	0.0038 (7)	0.0076 (7)	0.0033 (7)
O16	0.0255 (10)	0.0365 (10)	0.0443 (11)	−0.0035 (9)	0.0033 (9)	−0.0258 (9)
O17	0.0320 (10)	0.0435 (11)	0.0180 (9)	−0.0012 (9)	0.0083 (7)	0.0029 (8)
O18	0.0211 (9)	0.0311 (9)	0.0218 (9)	−0.0121 (7)	0.0063 (7)	−0.0029 (7)
C9	0.0201 (13)	0.0194 (11)	0.0221 (13)	−0.0008 (10)	0.0056 (10)	−0.0045 (10)
C10	0.0175 (12)	0.0181 (11)	0.0209 (12)	−0.0016 (10)	0.0026 (10)	−0.0042 (9)
C11	0.0205 (13)	0.0163 (11)	0.0224 (12)	−0.0006 (10)	0.0024 (10)	−0.0031 (9)
C12	0.0155 (12)	0.0197 (11)	0.0182 (12)	0.0048 (10)	0.0031 (9)	−0.0012 (9)
N1	0.0229 (11)	0.0232 (10)	0.0184 (10)	−0.0039 (9)	0.0031 (8)	0.0020 (8)
C13	0.0299 (14)	0.0409 (15)	0.0245 (13)	−0.0037 (13)	0.0102 (11)	−0.0016 (12)
C14	0.0382 (18)	0.0535 (19)	0.0481 (19)	0.0074 (16)	0.0106 (15)	−0.0084 (16)
C15	0.0286 (14)	0.0263 (13)	0.0241 (13)	−0.0029 (11)	0.0056 (11)	0.0057 (10)
C16	0.0345 (16)	0.0290 (14)	0.0396 (16)	−0.0059 (12)	0.0062 (13)	0.0088 (12)
C17	0.0288 (15)	0.0314 (13)	0.0280 (14)	−0.0014 (12)	−0.0036 (11)	−0.0045 (11)
C18	0.0387 (19)	0.0378 (17)	0.055 (2)	−0.0119 (14)	−0.0069 (16)	0.0025 (15)
C19	0.0285 (15)	0.0287 (13)	0.0297 (14)	−0.0048 (12)	−0.0043 (11)	0.0093 (11)
C20	0.0393 (18)	0.0340 (16)	0.062 (2)	0.0103 (14)	0.0003 (16)	0.0008 (15)
N2	0.0307 (12)	0.0308 (11)	0.0137 (9)	0.0042 (10)	0.0030 (9)	0.0012 (9)
C21	0.0364 (16)	0.0384 (15)	0.0208 (13)	0.0009 (13)	0.0051 (12)	−0.0062 (12)
C22	0.061 (2)	0.0441 (17)	0.0474 (19)	−0.0139 (16)	0.0234 (17)	−0.0142 (15)
C23	0.0292 (16)	0.0456 (16)	0.0279 (14)	0.0083 (13)	0.0110 (12)	0.0082 (12)
C24	0.042 (2)	0.0465 (18)	0.069 (2)	−0.0022 (16)	0.0227 (18)	−0.0028 (17)
C25	0.0346 (16)	0.0313 (14)	0.0204 (13)	0.0024 (12)	0.0096 (11)	−0.0024 (11)
C26	0.0419 (18)	0.0365 (15)	0.0369 (16)	0.0082 (13)	0.0154 (14)	0.0008 (13)
C27	0.0509 (19)	0.0422 (15)	0.0169 (13)	0.0139 (14)	−0.0031 (12)	0.0046 (12)
C28	0.054 (2)	0.0522 (19)	0.0235 (14)	0.0226 (17)	0.0008 (13)	0.0018 (14)
O1W	0.0376 (13)	0.0253 (10)	0.0444 (13)	−0.0045 (9)	0.0041 (10)	0.0029 (10)

### Geometric parameters (Å, °)

**Table:**

O1—C1	1.261 (3)	C14—H14B	0.9800
O2—C1	1.249 (3)	C14—H14A	0.9800
O3—C2	1.405 (3)	C15—C16	1.512 (4)
O3—H3O	0.8400	C15—H15A	0.9900
O4—C3	1.406 (3)	C15—H15B	0.9900
O4—H4O	0.8400	C16—H16C	0.9800
O5—C4	1.216 (3)	C16—H16B	0.9800
O6—C4	1.302 (3)	C16—H16A	0.9800
O6—H6O	0.8400	C17—C18	1.508 (4)
C1—C2	1.528 (3)	C17—H17A	0.9900
C2—C3	1.539 (3)	C17—H17B	0.9900
C2—H2	1.0000	C18—H18C	0.9800
C3—C4	1.519 (3)	C18—H18B	0.9800
C3—H3	1.0000	C18—H18A	0.9800
O7—C5	1.246 (3)	C19—C20	1.511 (4)
O8—C5	1.261 (3)	C19—H19A	0.9900
O9—C6	1.411 (3)	C19—H19B	0.9900
O9—H9O	0.8400	C20—H20A	0.9800
O10—C7	1.412 (3)	C20—H20B	0.9800
O10—H10O	0.8400	C20—H20C	0.9800
O11—C8	1.208 (3)	N2—C23	1.516 (3)
O12—C8	1.306 (3)	N2—C25	1.517 (3)
O12—H12O	0.8400	N2—C21	1.517 (3)
C5—C6	1.531 (3)	N2—C27	1.519 (3)
C6—C7	1.541 (3)	C21—C22	1.517 (4)
C6—H6	1.0000	C21—H21A	0.9900
C7—C8	1.517 (4)	C21—H21B	0.9900
C7—H7	1.0000	C22—H22C	0.9800
O13—C9	1.219 (3)	C22—H22B	0.9800
O14—C9	1.311 (3)	C22—H22A	0.9800
O14—H14O	0.8400	C23—C24	1.513 (5)
O15—C10	1.413 (3)	C23—H23A	0.9900
O15—H15O	0.8400	C23—H23B	0.9900
O16—C11	1.411 (3)	C24—H24C	0.9800
O16—H16O	0.8400	C24—H24B	0.9800
O17—C12	1.204 (3)	C24—H24A	0.9800
O18—C12	1.316 (3)	C25—C26	1.508 (4)
O18—H18O	0.8400	C25—H25A	0.9900
C9—C10	1.522 (3)	C25—H25B	0.9900
C10—C11	1.538 (3)	C26—H26C	0.9800
C10—H10	1.0000	C26—H26B	0.9800
C11—C12	1.520 (3)	C26—H26A	0.9800
C11—H11	1.0000	C27—C28	1.510 (4)
N1—C15	1.515 (3)	C27—H27A	0.9900
N1—C13	1.519 (3)	C27—H27B	0.9900
N1—C19	1.523 (3)	C28—H28C	0.9800
N1—C17	1.526 (3)	C28—H28B	0.9800
C13—C14	1.506 (4)	C28—H28A	0.9800
C13—H13A	0.9900	O1W—H1WA	0.83 (4)
C13—H13B	0.9900	O1W—H1WB	0.88 (5)
C14—H14C	0.9800		
			
C2—O3—H3O	109.5	H15A—C15—H15B	107.5
C3—O4—H4O	109.5	C15—C16—H16C	109.5
C4—O6—H6O	109.5	C15—C16—H16B	109.5
O2—C1—O1	126.1 (2)	H16C—C16—H16B	109.5
O2—C1—C2	115.9 (2)	C15—C16—H16A	109.5
O1—C1—C2	117.9 (2)	H16C—C16—H16A	109.5
O3—C2—C1	113.48 (19)	H16B—C16—H16A	109.5
O3—C2—C3	110.34 (19)	C18—C17—N1	115.5 (2)
C1—C2—C3	111.38 (19)	C18—C17—H17A	108.4
O3—C2—H2	107.1	N1—C17—H17A	108.4
C1—C2—H2	107.1	C18—C17—H17B	108.4
C3—C2—H2	107.1	N1—C17—H17B	108.4
O4—C3—C4	113.41 (19)	H17A—C17—H17B	107.5
O4—C3—C2	112.41 (19)	C17—C18—H18C	109.5
C4—C3—C2	108.57 (19)	C17—C18—H18B	109.5
O4—C3—H3	107.4	H18C—C18—H18B	109.5
C4—C3—H3	107.4	C17—C18—H18A	109.5
C2—C3—H3	107.4	H18C—C18—H18A	109.5
O5—C4—O6	124.8 (2)	H18B—C18—H18A	109.5
O5—C4—C3	124.2 (2)	C20—C19—N1	115.5 (2)
O6—C4—C3	111.1 (2)	C20—C19—H19A	108.4
C6—O9—H9O	109.5	N1—C19—H19A	108.4
C7—O10—H10O	109.5	C20—C19—H19B	108.4
C8—O12—H12O	109.5	N1—C19—H19B	108.4
O7—C5—O8	124.9 (2)	H19A—C19—H19B	107.5
O7—C5—C6	119.2 (2)	C19—C20—H20A	109.5
O8—C5—C6	115.8 (2)	C19—C20—H20B	109.5
O9—C6—C5	112.13 (19)	H20A—C20—H20B	109.5
O9—C6—C7	110.67 (19)	C19—C20—H20C	109.5
C5—C6—C7	109.97 (19)	H20A—C20—H20C	109.5
O9—C6—H6	108.0	H20B—C20—H20C	109.5
C5—C6—H6	108.0	C23—N2—C25	111.3 (2)
C7—C6—H6	108.0	C23—N2—C21	106.50 (19)
O10—C7—C8	111.96 (19)	C25—N2—C21	111.1 (2)
O10—C7—C6	111.45 (19)	C23—N2—C27	110.8 (2)
C8—C7—C6	109.8 (2)	C25—N2—C27	106.34 (19)
O10—C7—H7	107.8	C21—N2—C27	110.9 (2)
C8—C7—H7	107.8	C22—C21—N2	114.8 (2)
C6—C7—H7	107.8	C22—C21—H21A	108.6
O11—C8—O12	124.3 (2)	N2—C21—H21A	108.6
O11—C8—C7	123.0 (2)	C22—C21—H21B	108.6
O12—C8—C7	112.7 (2)	N2—C21—H21B	108.6
C9—O14—H14O	109.5	H21A—C21—H21B	107.5
C10—O15—H15O	109.5	C21—C22—H22C	109.5
C11—O16—H16O	109.5	C21—C22—H22B	109.5
C12—O18—H18O	109.5	H22C—C22—H22B	109.5
O13—C9—O14	125.2 (2)	C21—C22—H22A	109.5
O13—C9—C10	120.7 (2)	H22C—C22—H22A	109.5
O14—C9—C10	114.1 (2)	H22B—C22—H22A	109.5
O15—C10—C9	114.16 (19)	C24—C23—N2	115.5 (2)
O15—C10—C11	108.28 (18)	C24—C23—H23A	108.4
C9—C10—C11	108.58 (18)	N2—C23—H23A	108.4
O15—C10—H10	108.6	C24—C23—H23B	108.4
C9—C10—H10	108.6	N2—C23—H23B	108.4
C11—C10—H10	108.6	H23A—C23—H23B	107.5
O16—C11—C12	110.6 (2)	C23—C24—H24C	109.5
O16—C11—C10	111.75 (19)	C23—C24—H24B	109.5
C12—C11—C10	110.23 (18)	H24C—C24—H24B	109.5
O16—C11—H11	108.0	C23—C24—H24A	109.5
C12—C11—H11	108.0	H24C—C24—H24A	109.5
C10—C11—H11	108.0	H24B—C24—H24A	109.5
O17—C12—O18	125.1 (2)	C26—C25—N2	115.3 (2)
O17—C12—C11	124.2 (2)	C26—C25—H25A	108.4
O18—C12—C11	110.68 (19)	N2—C25—H25A	108.4
C15—N1—C13	111.18 (19)	C26—C25—H25B	108.4
C15—N1—C19	110.43 (19)	N2—C25—H25B	108.4
C13—N1—C19	106.50 (19)	H25A—C25—H25B	107.5
C15—N1—C17	106.36 (18)	C25—C26—H26C	109.5
C13—N1—C17	111.3 (2)	C25—C26—H26B	109.5
C19—N1—C17	111.15 (19)	H26C—C26—H26B	109.5
C14—C13—N1	115.5 (2)	C25—C26—H26A	109.5
C14—C13—H13A	108.4	H26C—C26—H26A	109.5
N1—C13—H13A	108.4	H26B—C26—H26A	109.5
C14—C13—H13B	108.4	C28—C27—N2	114.6 (2)
N1—C13—H13B	108.4	C28—C27—H27A	108.6
H13A—C13—H13B	107.5	N2—C27—H27A	108.6
C13—C14—H14C	109.5	C28—C27—H27B	108.6
C13—C14—H14B	109.5	N2—C27—H27B	108.6
H14C—C14—H14B	109.5	H27A—C27—H27B	107.6
C13—C14—H14A	109.5	C27—C28—H28C	109.5
H14C—C14—H14A	109.5	C27—C28—H28B	109.5
H14B—C14—H14A	109.5	H28C—C28—H28B	109.5
C16—C15—N1	115.1 (2)	C27—C28—H28A	109.5
C16—C15—H15A	108.5	H28C—C28—H28A	109.5
N1—C15—H15A	108.5	H28B—C28—H28A	109.5
C16—C15—H15B	108.5	H1WA—O1W—H1WB	108 (4)
N1—C15—H15B	108.5		
			
O2—C1—C2—O3	8.5 (3)	O15—C10—C11—C12	−67.9 (2)
O1—C1—C2—O3	−173.5 (2)	C9—C10—C11—C12	167.62 (19)
O2—C1—C2—C3	133.7 (2)	O16—C11—C12—O17	−10.4 (3)
O1—C1—C2—C3	−48.3 (3)	C10—C11—C12—O17	113.7 (3)
O3—C2—C3—O4	61.1 (2)	O16—C11—C12—O18	169.10 (19)
C1—C2—C3—O4	−65.8 (2)	C10—C11—C12—O18	−66.8 (2)
O3—C2—C3—C4	−65.2 (2)	C15—N1—C13—C14	60.4 (3)
C1—C2—C3—C4	167.88 (18)	C19—N1—C13—C14	−179.2 (2)
O4—C3—C4—O5	−7.3 (3)	C17—N1—C13—C14	−57.9 (3)
C2—C3—C4—O5	118.4 (2)	C13—N1—C15—C16	60.4 (3)
O4—C3—C4—O6	171.3 (2)	C19—N1—C15—C16	−57.6 (3)
C2—C3—C4—O6	−63.0 (2)	C17—N1—C15—C16	−178.3 (2)
O7—C5—C6—O9	165.8 (2)	C15—N1—C17—C18	179.2 (2)
O8—C5—C6—O9	−13.8 (3)	C13—N1—C17—C18	−59.5 (3)
O7—C5—C6—C7	−70.6 (3)	C19—N1—C17—C18	59.0 (3)
O8—C5—C6—C7	109.8 (2)	C15—N1—C19—C20	−54.8 (3)
O9—C6—C7—O10	63.3 (2)	C13—N1—C19—C20	−175.6 (2)
C5—C6—C7—O10	−61.1 (2)	C17—N1—C19—C20	63.0 (3)
O9—C6—C7—C8	−61.4 (2)	C23—N2—C21—C22	177.6 (3)
C5—C6—C7—C8	174.21 (18)	C25—N2—C21—C22	−61.1 (3)
O10—C7—C8—O11	−5.5 (4)	C27—N2—C21—C22	57.0 (3)
C6—C7—C8—O11	118.9 (3)	C25—N2—C23—C24	62.4 (3)
O10—C7—C8—O12	174.0 (2)	C21—N2—C23—C24	−176.4 (2)
C6—C7—C8—O12	−61.6 (3)	C27—N2—C23—C24	−55.7 (3)
O13—C9—C10—O15	168.0 (2)	C23—N2—C25—C26	60.9 (3)
O14—C9—C10—O15	−13.4 (3)	C21—N2—C25—C26	−57.6 (3)
O13—C9—C10—C11	−71.1 (3)	C27—N2—C25—C26	−178.4 (2)
O14—C9—C10—C11	107.5 (2)	C23—N2—C27—C28	−62.3 (3)
O15—C10—C11—O16	55.6 (2)	C25—N2—C27—C28	176.6 (3)
C9—C10—C11—O16	−68.9 (2)	C21—N2—C27—C28	55.7 (3)

### Hydrogen-bond geometry (Å, °)

**Table:**

*D*—H···*A*	*D*—H	H···*A*	*D*···*A*	*D*—H···*A*
O1W—H1WB···O2	0.89 (5)	2.56 (4)	2.995 (3)	111 (3)
O1W—H1WB···O5^i^	0.89 (5)	2.19 (5)	3.015 (3)	155 (4)
O3—H3O···O1W	0.84	2.04	2.868 (3)	167
O3—H3O···O2	0.84	2.29	2.645 (2)	106
O4—H4O···O5	0.84	2.51	2.778 (2)	100
O4—H4O···O11	0.84	1.99	2.790 (2)	160
O6—H6O···O2^ii^	0.84	1.65	2.485 (2)	175
O9—H9O···O1W^ii^	0.84	2.25	3.077 (3)	169
O9—H9O···O8	0.84	2.23	2.635 (2)	110
O10—H10O···O5	0.84	2.05	2.848 (2)	158
O10—H10O···O11	0.84	2.43	2.720 (3)	101
O14—H14O···O7^iii^	0.84	1.73	2.552 (2)	164
O15—H15O···O1	0.84	2.05	2.869 (2)	166
O16—H16O···O12^iii^	0.84	2.27	3.054 (3)	156
O18—H18O···O1^ii^	0.84	1.77	2.588 (2)	165
C2—H2···O6	1.00	2.45	2.852 (3)	103
C3—H3···O1	1.00	2.54	2.890 (3)	100
C3—H3···O15	1.00	2.55	3.503 (3)	158
C7—H7···O13^iv^	1.00	2.52	3.396 (3)	146
C10—H10···O18	1.00	2.56	2.920 (3)	101
C13—H13A···O1W^v^	0.99	2.51	3.419 (4)	152
C16—H16B···O3^vi^	0.98	2.46	3.378 (3)	155
C19—H19A···O3^vi^	0.99	2.38	3.275 (3)	150
C19—H19B···O9^vi^	0.99	2.37	3.294 (3)	155
C23—H23A···O17^vii^	0.99	2.47	3.420 (3)	161
C25—H25A···O13	0.99	2.46	3.419 (3)	164
C26—H26A···O9^viii^	0.98	2.56	3.482 (4)	157
C27—H27B···O18^i^	0.99	2.49	3.180 (3)	126

Symmetry codes: (i) *x*+1, *y*, *z*; (ii) *x*−1, *y*, *z*; (iii) −*x*+1, *y*+1/2, −*z*+1; (iv) −*x*+1, *y*−1/2, −*z*+1; (v) *x*−1, *y*, *z*+1; (vi) *x*, *y*, *z*+1; (vii) *x*+1, *y*, *z*−1; (viii) −*x*+1, *y*+1/2, −*z*.
